# Temporal course of implicit emotion regulation during a Priming-Identify task: an ERP study

**DOI:** 10.1038/srep41941

**Published:** 2017-02-02

**Authors:** Yi Wang, Xuebing Li

**Affiliations:** 1 CAS Key Laboratory of Mental Health, Institute of Psychology, Chinese Academy of Sciences, Beijing 100101, China; 2 University of Chinese Academy of Sciences, Beijing 100101, China

## Abstract

Implicit emotion regulation defined as goal-driven processes modulates emotion experiences and responses automatically without awareness. However, the temporal course of implicit emotion regulation is not clear. To address these issues, we adopted a new Priming-identify task (PI task) to manipulate implicit emotion regulation directly and observed the changes of early (N170), middle (early posterior negativity, EPN), and late event-related potentials (ERPs) components (late positivity potentials, LPP) under the different implicit emotion regulation conditions. The behavioral results indicated that the PI task manipulated subjective emotion experience effectively by priming emotion regulation goals. The ERP results found that implicit emotion regulation induced more negative N170 without altering the EPN and the LPP amplitudes, indicating that implicit emotion regulation occured automatically in the early perceptual stage not in the late selective attention stage of emotion processing. The correlation analysis also found the enlarged N170 was associated with decreased negative emotion subjective rating, suggesting that the N170 was probably an effective index of implicit emotion regulation. These observations imply that implicit emotion regulation probabbly occurs in the early stage of emotion processing automatically without consciousness.

In our daily life, people are surrounded by various life events evoking their emotions. The ability to control or express their emotions seems important to the quality of life and mental health. Unlike explicit emotion regulation which have to be conducted consciously with consumption of cognitive resources^1^, there is a growing focus on emotion regulation that operates at an implicit level without awareness or explicit instructions, labeled as automatic, unconscious or implicit emotion regulation^2^,^3^,^4^. Implicit emotion regulation is defined as goal-driven processes without conscious, deliberate control or awareness^1^,^5^. Koole and Rothermund^6^ emphasized the unintentional aspect and the lack of awareness as implicit emotion regulation. It turns out that implicit emotion regulation modulates negative emotion experience and physiological consequences, confirmed by several subsequent studies^7^,^8^,^9^,^10^.

Previous researches emphasized the goal-directed nature of implicit emotion regulation^6^. Williams and his colleagues found that nonconsciously operating emotion regulation goals by priming reappraisal emotion control goals decreased the heart rate as comparing with those explicitly instructed to reappraise, highlighting the potential importance of nonconscious goal-pursuit in implicit emotion regulation^8^, which findings indicated that the implicit emotion regulation processes may be induced by the activated relevant emotion regulation goals^11^. According to an action control perspective^12^, the implicit processes may support emotion regulation in three ways. Firstly, implicit processes may make people to decide whether or not to regulate emotions by implicitly activating the emotion regulation goals and monitoring the emotional responses to be compatible with regulation goals. Secondly, implicit processes may activate habitual strategies and adjust strategies to situational affordances. Thirdly, the recruited implicit processes may facilitate the enactment of emotion regulation strategies. Thus the mechanism underlying implicit emotion regulation is posited to be nonconscious goal pursuit^8^.

Despite the wealthy evidence showed that implicit emotion regulation modulates emotion experience, the neural mechanism of implicit emotion regulation remains unclear so far. Theories indicated that implicit emotion regulation operates quickly and automatically in the emotion trajectory^3^, making it hard to investigate its time course during emotion processing. Event-related potentials (ERPs), a high temporal resolution technique, is effective to study the time course of implicit emotion regulation^13^. To investigate these aspects in detail, there are some ERP studies have been conducted. Zhang and Zhou^14^ required participants to view emotional pictures to examine the individual differences of automatic emotion regulation. The results showed that the automatic emotion express group rather than the automatic emotion control group showed larger late positivity potential (LPP) amplitudes in the right hemisphere. Mocaiber *et al*.^15^ found that using an “implicit reappraisal strategy” attenuated the reaction time and the amplitudes of the early window of LPP while viewing affective pictures. Zhang and Lu^16^ adopted a modified facial Go/Nogo paradigm to examined the time course of automatic emotion regulation processing, in which participants made only a gender judgment of emotional faces. They found both Go-N2 and Nogo-P3 components could be modulated by automatic emotion regulation. Another ERP study investigated the influence of implicit emotion regulation on outcome evaluation to gains and losses, which found that priming emotion regulation reduced the amplitude of the feedback-related negativity (FRN) rather than the P300, reflecting that implicit emotion regulation could modulate outcome evaluation effectively without costing cognitive resources^9^. These ERP studies of implicit emotion regulation have shown some significant and interesting results, but there are still some questions remaining unsolved. Firstly, implicit emotion regulation was defined as goal-driven changes to any aspect of emotion without conscious, attention, or deliberate control^3^,^17^, but the experimental tasks adopted by those studies did not involve or manipulate goal pursuit aspects, which might not reflect the implicit emotion regulation processes. Secondly, FRN, N2, and P300 (LPP) are all middle or late ERP components^9^,^18^. Since the implicit emotion regulation operates automatically without awareness^6^, it might be indexed by the earlier ERP components associated with the automatic processing.

Accordingly, in the current study, we developed a new ERP paradigm named Priming-Identify task (PI) manipulating implicit emotion regulation as an independent variate. The PI task included a priming phase and an identify phase. During the priming phase, participants were required to perform word matching tasks which they were primed with different emotion regulation goals unconsciously (e.g. emotion control, emotion expression). During the subsequent identify phase, participants were asked to recognize facial expressions to investigate the time course of implicit emotion regulation under emotion regulation goals.

Growing evidences show that a priming technique could effectively activate and manipulate emotion regulation goals without consciousness^19^, leading to attenuated emotion experience and physiological responses like heart-rate reactivity or electrophysiology responding^8^,^20^. The activated emotion regulation goals may then induce implicit emotion regulation processing and lead to emotion experiences and responses^8^,^21^. Emotion control and emotion expression are two typical emotion regulation goals in the previous studies^3^. Thus in the present study, we employed a word matching task before the emotion processing to activate the emotion regulation goals implicitly, by priming emotion regulation goals (emotion control, emotion expression).

We adopted a facial expression identify task to investigate the temporal course of implicit emotion regulation during facial emotional processing. Since the emotion regulation processes may have their effects in the emotion generative process^22^, it is difficult to separate the regulation processing from the whole emotion processing. Thus we investigated the time course of implicit emotion regulation by manipulating the emotional processing with activated emotion regulation goals. The ERP-related time course of emotion processing can be summarized into three stages as early stage (100–200 ms), middle stage (200–300 ms), and late stage (>300 ms) based on the latency of ERP components^23^. According to previous studies on the time course of facial processing^24^,^25^,^26^, we chose three ERP components to examine the modulation effects of implicit emotion regulation: N170, early posterior negativity (EPN), and late positivity potentials (LPP), indicating the three stages of emotion processing respectively.

N170 is a negative deflection located at the occipito-temporal regions that peaks at around 170 ms post stimulus, with right-hemisphere dominance. Facial stimuli induced enhanced N170 amplitudes compared to non-facial stimuli^27^. Therefore, N170 is regarded as a face-specific ERP component^28^, interpreted as an early perception process of the structural encoding of faces^29^, and not affected by top-down attention control, with automatic processing properties^27^. However, it is debatable whether the N170 is modulated by different emotional expressions of a face, as will be described later. EPN is a negative-going waveform at around 150–300 ms after the stimuli onset over occipito-temporal regions^18^. It has been consistently demonstrated that the EPN is the first cortical ERP component which reflect the selective processing of emotion^30^. The amplitude of EPN was enlarged to emotional stimuli, especially threatening stimuli^24^,^31^. The early window of LPP is a centro-parietal positivity peaking at 300 ms or later after stimulus onset with latency at around 300–700 ms. According to previous studies, the early window of LPP increases in response to emotionally salient stimuli, which represents sustained attention resources are allocated to emotional stimuli^9^,^32^. In addition, the middle and late ERP components such as EPN and LPP were mostly indicators of the conscious cognitive resources allocation in emotional processing, and the early ERP component (N170) was thought to be automatic and independent of top-down attention control^13^,^15^,^18^.

Based on the literatures above, the aim of the present study was to investigate the time course of implicit emotion regulation during facial emotional processing with the new PI task. For the threatening emotions such as anger and fear^33^ played an important role in self-regulation, the current study focused on the time course of implicitly regulating threatening emotions. According to the implicit emotion regulation researches using priming technique, we hypothesized that the activated emotion regulation goals would reduce subjective experience of negative emotions in the subsequent emotion processing. Since implicit emotion regulation usually occurs automatically without deliberate control or awareness, we predicted that the priming goals probably influence the early perception processing stage (N170) which is automatic and costs less cognitive resources, but did not affect the middle or late ERP components (EPN and LPP) requiring high-level cognitive processes and more cognitive resources.

## Results

### Subjective ratings and behavioral performance

One of twenty-three participants realized the word matching task aimed to regulate negative emotion in the following task, and this subject’ data have been excluded in all subsequent analyses.

**Figure 1:**
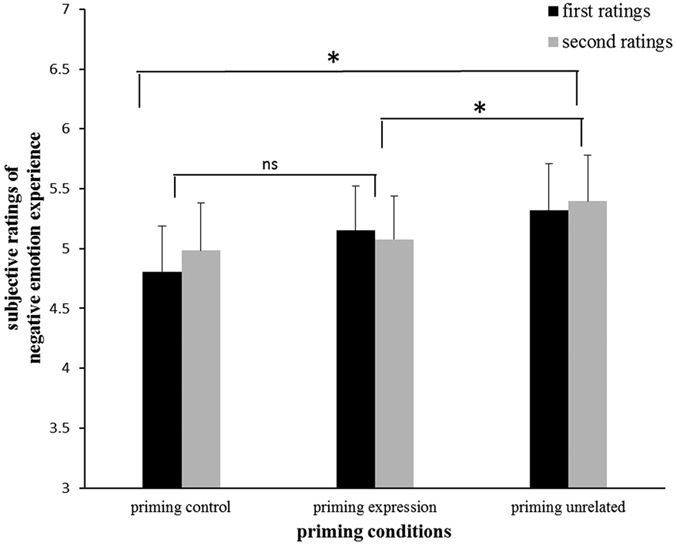
The subjective ratings of negative emotion experience in three priming conditions and two ordinal positions. The larger rating scores indicated stronger negative emotion experience. The ANOVAs results revealed a main effect of priming conditions. Both priming control and expression showed lower negative emotion experience ratings than priming unrelated condition, regardless of the ordinal positions. The difference between priming control and expression was not significant. Error bars indicate the standard error (* means *p* < 0.050).

Negative emotion rating scores were entered into a 3 (priming condition: control/expression/unrelated) × 2 (ordinal positions: first/second) repeated measures ANOVAs. The results revealed a significant main effect of priming conditions (*F*_(*2, 42*)_ = 4.518, *p* = 0.033, *η*_*p*_^*2*^ = 0.177). Both priming control (*p* = 0.029) and expression (*p* = 0.019) showed lower negative emotion experience ratings than unrelated condition (see Fig. 1). The difference between priming control and expression was not significant (*p* = 0.152). The main effect of ordinal positions (*p* = 0.450) and the interaction between priming conditions and ordinal positions (p = 0.101) were not significant.

For the reaction time of emotion distinction task, the difference of priming conditions (priming control, priming expression, unrelated) was not significant (*p* = 0.227). For accuracy of the emotion distinction task, the difference of priming conditions was also not significant (*p* = 0.110). The descriptive statistical results of reaction time and accuracy are presented in Table. 1.

### ERP results

#### N170

**Figure 2:**
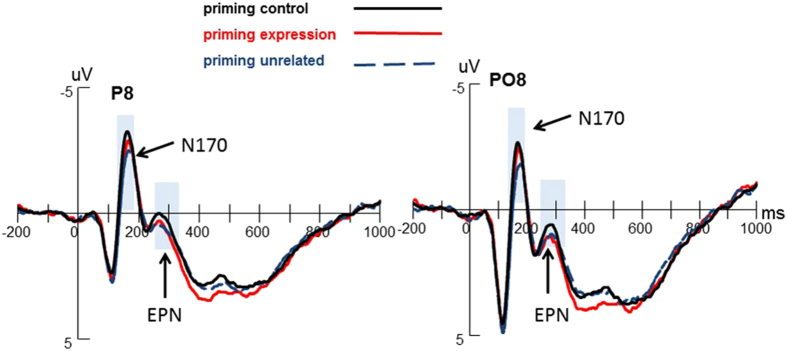
Grand averages of N170 and EPN amplitudes evoked during the PI task at P8 and PO8. Priming emotion regulation goals enhanced N170 amplitude compared to priming unrelated words, whereas EPN amplitude was not modulated by implicit emotion regulation.

The results of a 3 (priming conditions) × 2 (horizontal electrodes: P/PO) × 4 (vertical electrodes: 5/6/7/8) ANOVAs revealed significant main effects of priming conditions (*F*_(*2, 42*)_ = 3.856, *p* = 0.029, *η*_*p*_^*2*^ = 0.155), horizontal electrodes (*F*_(*1, 21*)_ = 14.031, *p* = 0.001, *η*_*p*_^*2*^ = 0.401), and vertical electrodes (*F*_(*3, 63*)_ = 9.945, *p* = 0.001, *η*_*p*_^*2*^ = 0.321). For priming conditions main effect, a subsequent post hoc test showed that both priming control (*p* = 0.006) and priming expression (*p* = 0.044) induced more negative N170 amplitudes than unrelated condition. The difference between priming control and expression was not significant (*p* = 0.459) (see Fig. 2). The main effects of electrodes showed that the parietal sites elicited more negative N170 amplitudes than the parietal-occipital sites, and the N170 at P8 and PO8 were significantly larger than other vertical electrode sites.

#### EPN

3 (priming conditions) × 2 (horizontal electrodes) × 4 (vertical electrodes) repeated measures ANOVAs was conducted for EPN amplitudes. The main effect of priming conditions (*F*_(*2, 42*)_ = 1.926, *p* = 0.158) was not significant. We found a significant main effect of vertical electrodes (*F*_(*3, 63*)_ = 6.027, *p* = 0.011, *η*_*p*_^*2*^ = 0.223). The right hemisphere sites elicited more negative EPN than the left hemisphere sites. Other main effects or interactions were not significant (see Fig. 2).

We controlled age and gender as covariate and conducted the ANOVAs analysis again. The main effects of the priming conditions (*F*_(*2, 38*)_ = 2.40, *p* = 0.104), horizontal electrodes (*F*_(*1, 19*)_ < 0.01, *p* = 0.994), vertical electrodes (*F*_(*3, 57*)_ = 0.82, *p* = 0.486), and the interactions among variables (*ps* > 0.05) were not significant.

#### LPP

**Figure 3:**
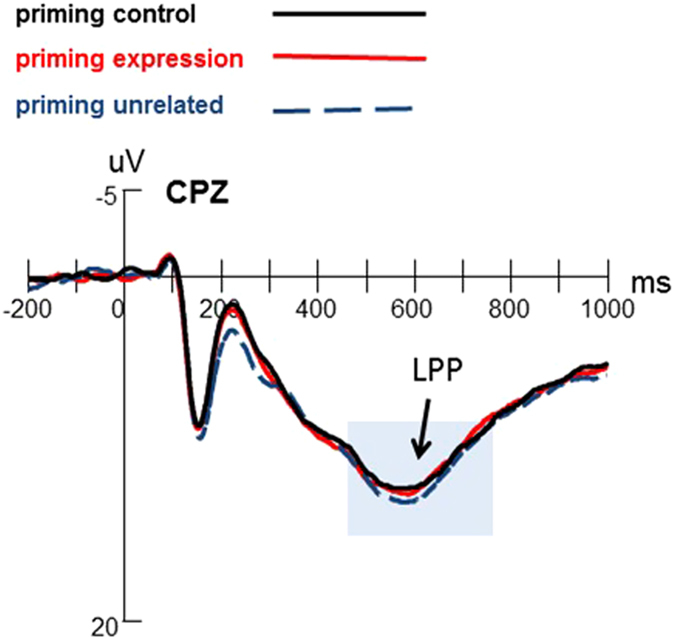
Grand averages of LPP elicited by threatening faces during the PI task at CPZ. The LPP amplitude did not vary with priming conditions.

The amplitudes of LPP were entered into 3 (priming conditions) × 3 (vertical electrodes) × 3 (horizontal electrodes) repeated measures ANOVAs. The results showed significant main effects of vertical electrodes (*F*_(*2, 42*)_ = 27.889, *p* < 0.001, *η*_*p*_^*2*^ = 0.57) and horizontal electrodes (*F*_(*2, 42*)_ = 3.828, *p* = 0.030, *η*_*p*_^*2*^ = 0.154). The post hoc test of the main effect of vertical electrodes showed a decrease from midline to right locations (*p* < 0.001) and from right to left locations (*p* < 0.001). The central-parietal sites induced largest LPP than central and parietal sites. The effect of priming conditions was not significant (*F*_(*2, 42*)_ = 0.391, *p* = 0.679) (see Fig. 3).

Since age and gender might affect the amplitudes of LPP^34^, we controlled age and gender as covariate and conducted the ANOVAs analysis again. The main effects of priming conditions (*F*_(*2, 38*)_ = 0.29, *p* = 0.750), horizontal electrodes (*F*_(*2, 38*)_ = 0.56, *p* = 0.578), vertical electrodes (*F*_(*2, 38*)_ = 1.90, *p* = 0.164), and the interactions among variables (*ps* > 0.05) were not significant.

### Correlations

**Figure 4:**
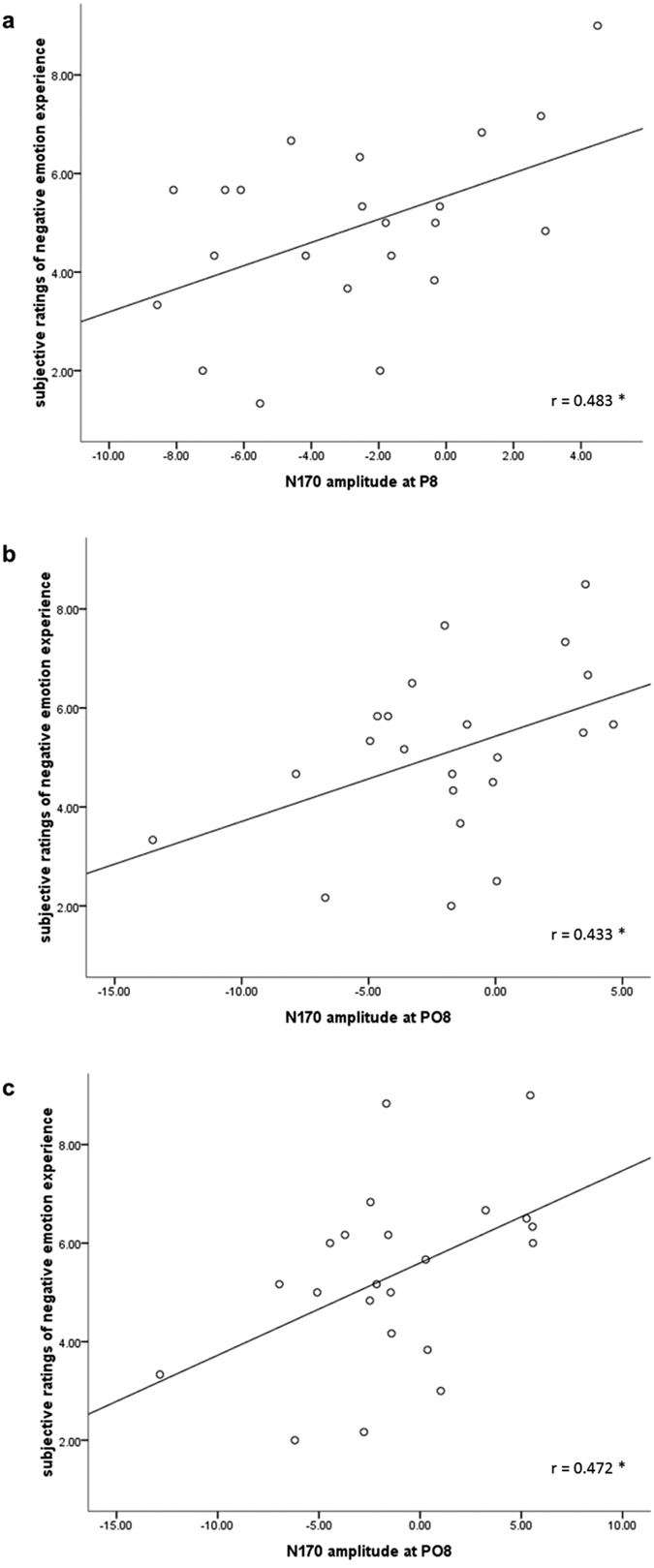
Two-tailed correlations between N170 amplitude and emotion ratings of negative emotion experience. (**a**) priming control condition (**b**) priming expression condition (**c**) priming unrelated condition.

To examine the relationship between subjective emotional experiences and the changes of N170 amplitudes, Pearson (*r*) correlation coefficients were computed between the subjective rating scores and the value of N170 amplitudes at P8 and PO8 where the N170 was mostly elicited for the three priming conditions respectively. We have examined the time effect of subjective ratings, and the ANOVA analysis showed that the main effect of ordinal positions was not significant (*p* = 0.450). Thus we used the averages of the twice subjective ratings as the index of subjective emotional experience. The results showed that the decreased negative emotion ratings were associated with a larger N170 in priming control condition (P8: *r*_(*22*)_ = 0.483, *p* = 0.023; PO8: *r*_(*22*)_ = 0.421, *p* = 0.051), priming expression condition (P8: *r*_(*22*)_ = 0.404, *p* = 0.062; PO8: *r*_(*22*)_ = 0.433, *p* = 0.044), and unrelated condition (P8: *r*_(*22*)_ = 0.418, *p* = 0.053; PO8: *r*_(*22*)_ = 0.472, *p* = 0.027). The scatter diagrams in the significant electrode sites were showed in Fig. 4.

## Discussion

The present study characterized the time course of implicit emotion regulation with activated emotion regulation goals. Consistent with our hypothesis, we found a priming effect of subjective emotional ratings: priming emotion control and expression reduced negative emotion experience. ERP results showed significant priming effects for the early stage component, and increased N170 was observed when priming emotion control and emotion expression, as compared with priming unrelated words. On the contrary, the middle and late stages processing (EPN, LPP) were not modulated by implicit emotion regulation goals. In addition, this enhanced N170 was associated with reduced subjective ratings of negative emotion experience.

The emotion regulation goal-pursuit has been demonstrated to be a form of implicit emotion regulation occurring without awareness^1^,^35^. In the present study, the priming task activated goals in the subsequent facial emotional processing unconsciously, leading to reduced subjective emotion experience with modulated ERP components in emotion processing, which confirmed our hypothesis and the extended process model of emotion regulation (EMP). In the perspective of EPM, emotion regulation must be something to achieve a particular goal, rather than a passive response to a specific emotion state^11^. In the current PI task, the priming task was designed as a word matching task irrelevant to the emotion categorization task. This manipulation ensured the goal pursuit was executed implicitly. In our study, only one participant of twenty-three realized the word matching task aimed to regulate negative emotion in the following task. We then compared the ordinal position of rating scores and found no difference between the first and the second rating after the priming task, illustrating that the priming effect was not attenuated with time. These two results together indicate that the manipulation of implicit emotion regulation in the present study was valid as well as durable.

The subjective rating results revealed that both priming emotion control and priming emotion expression decreased negative experience as compared to unrelated words, which is partially consistent with a previous study^3^. In Mauss *et al*.’s study, there were only two conditions (priming emotion control and priming emotion expression) to compare, and they found priming emotion control reduced subjective emotion experience more than priming emotion expression. In the current study, we adopted both emotion control and emotion expression as priming emotion regulation conditions compared to priming unrelated words. Although emotion control showed a trend of reducing negative ratings compared with emotion expression (see Fig. 1), the added unrelated condition might weaken statistical difference of these two regulation conditions. In addition, emotion control words and emotion expression words used in Mauss’s study were paired in the same sentence reflecting the opposite meanings. However, in our study, the emotion control words (e.g. control, restrain) have the meaning of inhibiting emotion, directly suggesting the participants to down-regulate their emotions; by contrast, the words of emotion expression in Chinese (e.g. express, reveal) have the meaning of simply letting others know your feelings, and do not specifically direct participants to up-regulate emotions. This might be another reason that no difference between emotion control and emotion expression was found.

More interesting finding in the present study is that priming emotion control and emotion expression enlarged N170 amplitudes in the subsequent facial emotion processing, as comparing with priming unrelated words, which supported our hypothesis that the priming goals would influence the early perception processing stage (N170) which is automatic and costs less cognitive resources. Correlation results also showed the subjective negative emotion experience reduced with the enlargement of N170 amplitudes, which is probably due to the structure encoding process of faces had been deepened by implicit emotion regulation goals. As mentioned above, N170 is considered to be a marker of facial processing reflecting the encoding of the configuration of faces^36^,^37^,^38^. According to the face processing model proposed by Bruce and Young^39^, the structure encoding unit includes the view-center descriptions module and the expression-independent descriptions module, which might be isolated from each other. The view-center descriptions module provides information for the analysis of expression, and the expression-independent descriptions module provides information for the global configuration and of features. Previous studies have indicated that facial stimuli induced enhanced N170 amplitudes compared to non-facial stimuli^40^, and a number of studies found no significant N170 differences between emotional expressions (including happy, fear, anger, disgust) and neutral ones^41^,^42^,^43^, thus the larger N170 in this study should imply better structural encoding of faces.

Notably, although the idea of the N170 insensitivity to emotional faces is still relatively solid^44^, enhanced N170 amplitudes elicited by different facial expressions have been also reported in several studies^45^,^46^. For instance, Rellecke *et al*.^45^ asked participants to process happy, angry and neutral faces in an explicit or implicit task condition, and found the N170 amplitudes of happy and anger expressions were greater than neutral expression under the both task conditions. Pegna *et al*.^46^ found that even if the faces were presented under the perception threshold, N170 amplitudes of happy or fear expressions were still greater than the neutral ones. However, the contribution of potential moderators that may account for these divergent results regarding the sensitivity of the N170 to facial expression is still not clear. For all this, in the current study face expressions were homogeneous among all three compared experimental conditions, and then the enhanced N170 could not reflect more elaborate emotion recognition processing. Therefore, the priming effect of N170 amplitudes in the current study was suggested that emotion regulation goals deepen the processes of emotion-unrelated facial structural encoding, facilitating the facial structural encoding. Similarly, Tupak and his colleagues adopted a cognitive labeling paradigm to investigate the neural activations of implicit emotion regulation and their results indicated that guiding attention to non-emotion aspects instead of affective aspects of stimuli produced increased activations in brain regions associated with emotion regulation^47^. At last, N170 is a component at the early stage of emotion processing^18^. And a meta-analysis study found that the N170 elicited by facial expression is modulated by automatic processes that do not require awareness^37^. Thus, the altered N170 in this study suggests that the implicit emotion regulation may occur very early in facial perception processing without consciousness.

During the middle and late stages of emotion processing, there were no priming effects observed for EPN and LPP components. In facial emotion processing, faces are usually detected at N170, further emotional encoding at EPN, and elaborate processing at LPP^23^,^24^. Specifically, the middle and late ERP components (such as EPN and LPP) were well known to require more cognitive resources and attention control than the early ERP components (such as N170)^13^,^18^,^48^. Previous studies have observed significant emotional effects on EPN, showing that the emotional stimuli induced a greater EPN than the neutral stimuli, no matter the stimuli are scenes or faces^31^,^46^,^49^. The widespread emotion effect of EPN could reflect that the visual cortex gives further selective attention to the emotional information from environment after the perception encoding process^50^,^51^. Whilst, many studies have suggested that the LPP is an index of cognitive demands, representing the attention resources allocation during task processing, and the early window of LPP indicated that sustained attention resources are allocated to emotional stimuli^14^,^52^. No significant EPN and LPP differences among the three priming conditions in our data implied that the implicitly activation of emotion regulation goals could not modulate the selective attention process and the cognitive resources allocation during the facial emotion processing. In contrast to explicit emotion regulation which costs cognitive resources when adopting instructed strategies^53^,^54^,^55^, implicit emotion regulation is usually automatic and costs less cognitive resources. Mauss, Cook, *et al*.^3^ found that priming emotion control reduced negative emotion experience without the changed cardiovascular responding. Similarly, Yang *et al*. did not observe the priming emotion regulation reduced the amplitude of P300 and they suggested that implicit emotion regulation could modulate outcome evaluation effectively without costing cognitive resources^9^. González-Garrido investigated the effects of implicit facial emotional processing on the object-discrimination ability and did not find differences of LPP amplitudes among different facial emotion contexts (e.g. neutral, happy, fear), indicating that the LPP amplitude was unaffected by implicit emotion processing^56^.

However, some other studies found the inconsistent results of the late ERP components with the present study^14^,^15^,^16^. For instance, Zhang’s study employed the facial Go/Nogo task and found Nogo-P3 was reduced under the emotional conditions compared with the neutral condition^16^. On one hand, Nogo-P3 represents different functional meaning (response inhibition) from LPP (emotion experience); on the other hand, the modulated P3 was observed between the emotional condition and the neutral one not between the emotion regulation conditions, which probably caused the inconsistent results with the present study. In Mocaiber’s study, unpleasant pictures were presented as distractors, competing for attention with the primary task demands, the decreased LPP may be the results of the interplay between implicit regulation of negative emotions and the main cognitive task (the bar-orientation task)^15^. However, in the present study, implicit emotion regulation was the only variate among different experimental conditions, excluding the effects of other cognitive activities on the implicit emotion regulation processes. Therefore, the results of EPN and LPP in our study probably reflected that implicit emotion regulation does not cause conscious processes or more cognitive resources occupation in middle and late stages of emotion processing.

A few limitations of the current study should be addressed. Firstly, we could not compare implicit down-regulation and implicit up-regulation, because the emotion expression words we used did not direct the participants to up-regulate their feelings. To directly compare the effects of up-regulating goals and down-regulating goals, future research could use words that advise people to up-regulate their emotions instead of emotion expression words. Secondly, since the present study did not contain the neutral faces under the priming unrelated condition, it is uncertain that the threatening faces in the present study induced enough threatening emotions in the emotion distinction task as we assumed. Thirdly, the participants were required to report their subjective emotion experiences after the whole threatening emotion regulation processing, disregarding the valance differences and the topographical structural processing differences between anger and fear. Further studies should focus on specific implicit emotion regulation of anger or fear.

Taken together, the results of this study revealed that priming emotion control and emotion expression goals effectively reduced subjective negative experience. Pursuing emotion regulation goals elicited larger N170 amplitude, which was correlated to the reduced subjective emotion experience. In contrast, EPN and LPP amplitudes elicited by emotional faces were not modulated by implicit emotion regulation goals. These results suggest that implicit emotion regulation occurs at very early perceptual stage of emotion processing and the N170 is probably an effective index of implicit emotion regulation. The current study provides a clear insight into the neural mechanism and the time course of implicit emotion regulation during emotion processing, and is probably the first direct evidence to confirm the theory that implicit emotion regulation occurs automatically without awareness^6^.

## Methods

### Participants

Twenty three healthy adults in Beijing, China participated in our experiment. All of them were right-handed, were mentally healthy, and had normal or corrected to normal vision (9 male, mean age 23.60 years, *SD* = 1.98). The research was approved by the ethics committee of the Institute of Psychology, Chinese Academy of Sciences and was carried out in accordance with the approved guidelines. All participants provided written informed consent before the experiment and received a small payment as compensation.

### Stimuli

#### Priming words

Twenty students were required to categorize 100 Chinese two-character verbs into three categories according to their meaning: emotion control, emotion expression, or unrelated to either. The 40 words on the final list were those that had been placed into one category by at least 15 students. The categories of “control” (e.g. adjust, inhibit) and “expression” (e.g. release, express) had 10 words respectively, and the category of “unrelated” (e.g. cancel, run) had 20 words. The words we used were displayed in Supplementary Information 1. Another 20 students rated the 40 selected words on valence, arousal, familiarity, and how they related to emotion control and emotion expression with a 7-point scale. One-way ANOVAs showed no difference among categories on valence (*p* = 0.337), arousal (*p* = 0.183), or familiarity (*p* = 0.126). “Control” words were rated significantly higher than “expression” (*p* < 0.001) and “unrelated” words (*p* < 0.001) on their relatedness to emotion control. “Expression” words were rated significantly higher than “control” (*p* < 0.001) and “unrelated” words (*p* < 0.001) on their relatedness to emotion expression. “Control” words were rated higher on relatedness to emotion control than to emotion expression (*p* < 0.001). “Expression” words were rated higher on relatedness to emotion expression than to emotion control (*p* < 0.001).

#### Faces

The formal experiment consisted of eighty pictures of faces selected from the Chinese Facial Affective Picture System (CFAPS)^26^, including 40 fear faces and 40 anger faces. The numbers of pictures we used were displayed in Supplementary Information2. Male and female faces were equally presented in these two emotions. The difference of arousal between fear and anger faces based on the data of the CFAPS data base was not significant (*t*_(*78*)_ = −0.94, *p* = 0.351). Each picture was adjusted to the same appropriate brightness and contrast. Subjects were seated in a quiet room with eyes 60 cm from the screen, the viewing angle was 4.2 × 5.1°, and each picture was presented in the center of the screen.

### Experiment design and procedure

The formal PI paradigm contained a word matching task and a facial expression identify task. The word matching task had three conditions according to the priming word categories (i.e., “control,” “expression,” and “unrelated”). The facial expression identify task had two facial expressions, i.e. anger and fear. Participants were told that the aim of the present study was to investigate the vocabulary processing and the emotional processing. The formal experiment contained a word matching task and a facial expression identify task conducting alternatively. Participants were required to complete these two tasks following their corresponding instructions.

During the word matching task, three words were presented with one on the top of the screen and two at the bottom of the screen. Participants were required to choose a word at the bottom to match the meaning of the word on the top by pressing “O” for the left-side word and “P” for the right-side word. In the control condition, two of the three words in each trial belonged to the category of “control” and the other belonged to the category of “unrelated”. In the expression condition, two of the three words in each trial belonged to the category of “expression” and the other belonged to the “unrelated” category. In the unrelated condition, all three words belonged to the “unrelated” category. Participants were told that this task was aimed at checking their vocabulary ability.

During the facial expression identify task, a fixation was presented at the center of the screen for 200–500 ms. A facial picture was then presented for 1000 ms. Participants were required to judge whether the expression was anger or fear as soon as possible by pressing “K” or “L”. The corresponding relation between emotion and button was balanced across subjects. After the picture disappeared, a 1000 ms blank was present between trials. During and after the emotion categorization task, subjects were asked to rate their negative emotion experience level with a 9-point scale, every 30 trials.

**Figure 5:**
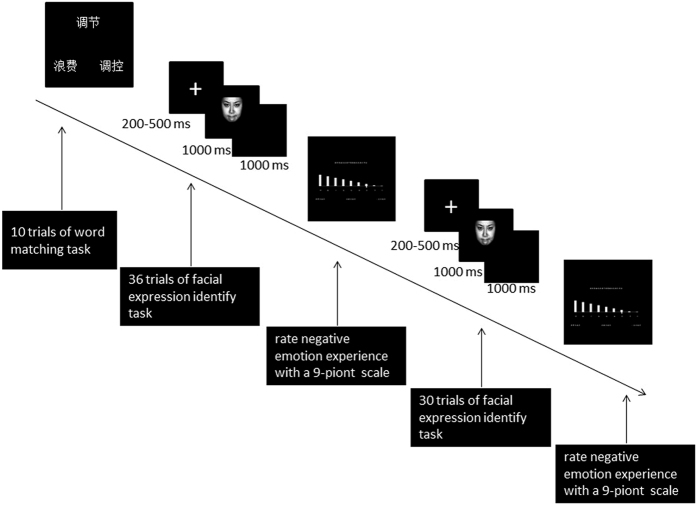
The sequence of the Priming-Identify task in one single block. Participants were required to complete 10 trials of word matching task, followed by 66 trials of emotion distinction task, with negative emotion experience ratings twice. The facial materials in the emotion distinction task were chosen from the Chinese Facial Affective Picture System (CFAPS).

The order of the three priming conditions was balanced across subjects. Each priming condition was divided into three successive blocks. In each block, subjects were asked to finish 10 word matching trials, followed by 66 expression categorization trials and two ratings of negative emotion (see Fig. 5). The expression identify task in each block consisted of anger and fear faces, 33 each. Thus the whole experiment consisted of 90 trials (3 priming conditions × 3 blocks × 10 trials) of the word matching task, 594 trials (3 priming conditions × 3 blocks × 2 emotions × 33 trials) of emotion categorization task, and 18 negative emotion ratings (3 priming conditions × 2 ordinal positions × 3 repetition). The first 6 trials of the expression identify task in each block were excluded from data analysis in order to minimize the effects of distraction due to task switching. There were 540 expression identify trials used in the data analysis.

After all of these tasks, participants were asked to report whether they saw a relationship between the word matching task and the expression identify task. Those who realized the priming function of the word matching task were excluded from the final data analysis, since their emotion regulation processes were not operated implicitly.

### EEG recording

Electrical activity during the emotion distinction task was recorded with 64 electrodes placed on an elastic scalp (NeuroScan 4.5). The vertical electrooculogram (VEOG) was recorded above and below the left eye. The horizontal electrooculogram (HEOG) was recorded as the left versus right orbital rim. The electroencephalogram (EEG) activity was recorded using a left mastoid reference electrode and re-referenced off-line to the mean of bilateral mastoid electrodes. All electrode impedances were maintained below 5 KΩ. EEG and EOG were amplified using a 0.05–100 Hz band-pass and sampled at the rate of 1000 Hz. The EEG data were filtered offline with a low-pass filter at 30 Hz (12 dB/oct). The ERP data were segmented for each condition from −200 ms to 1000 ms relative to the face picture onset in the emotion distinction task, with a baseline correction 200 ms pre-stimulus. Trials containing activity exceeding ± 80 μV at any site were excluded from averaging. At least 70 effective trials each condition were left after artifact-removing, and the differences among three priming conditions for the number of left effective trials after removing artifacts were not significant (*p* = 0.957).

### Data analysis

We analyzed subjects’ ratings for three priming conditions (control, expression, unrelated) and two ordinal positions (first, second). For behavioral data, we calculated the reaction time of the facial expression identify task for correct trials in three priming condition (control, expression, unrelated). Trials were excluded if the reaction time was shorter than 200 ms or longer than 1500 ms.

For ERP data, average amplitudes were overlaid for correct trials in the three priming conditions in the facial expression identify task. Since there were no significant differences of priming effects between fear and anger face processing, we combined fear and anger faces together. In our study, N170, EPN, and LPP were measured and analyzed for averaged amplitudes. According to previous ERP studies^24^,^26^, eight electrode sites (P5, P6, P7, P8, PO5, PO6, PO7, and PO8) were selected for N170 (145–190 ms) and EPN (250–320 ms) analysis. LPP was described as a late centro-parietal component during emotion processing^24^,^31^, nine electrode sites (C3, CZ, C4, CP3, CPZ, CP4, P3, PZ, P4) were selected for analysis of LPP (450–750 ms).

The Greenhouse-Geisser correction was used to compensate for sphericity violations. Least significant difference (LSD) was applied for post hoc testing of main effects. Partial eta-squared (*η*_*p*_^2^) was reported as indicator of the effect size in ANOVA tests. All these statistical analysis were conducted with SPSS 17.0 software.

## Additional Information

**How to cite this article**: Wang, Y. and Li, X. Temporal course of implicit emotion regulation during a Priming-Identify task: an ERP study. *Sci. Rep.* **7**, 41941; doi: 10.1038/srep41941 (2017).

**Publisher's note:** Springer Nature remains neutral with regard to jurisdictional claims in published maps and institutional affiliations.

## Supplementary Material

Supplementary Information

## Figures and Tables

**Table 1:** Descriptive statistics of reaction time and the accuracy for priming conditions (n = 22).

		Reaction time		accuracy	
		Mean (ms)	SD	Mean (ms)	SD
Priming conditions	Control	716.71	76.65	0.86	0.05
	Expression	729.51	80.00	0.84	0.06
	Unrelated	721.06	68.18	0.86	0.06
