# Supplementary material for: Temporal course of implicit emotion regulation during a Priming-Identify task: an ERP study
Source: Sci Rep. 2017 Feb 2;7:41941. doi: 10.1038/srep41941 (PMC5288784; doi:10.1038/srep41941)
Supplement: Supplementary Information [file srep41941-s1.doc]

**Temporal course of implicit emotion regulation during a Priming-Identify task: an ERP study**

Yi Wang^1,2^ and Xuebing Li^1*^

^1^ Neuropsychology and Applied Cognitive Neuroscience Laboratory, Key Laboratory of Mental Health, Institute of Psychology, Chinese Academy of Sciences, Beijing 100101, China

^2^ University of Chinese Academy of Sciences, Beijing 100101, China

**Supplementary Information**

**1. Words used in the word watching task**

| control | | express | | unrelated | |
| --- | --- | --- | --- | --- | --- |
| Chinese | English | Chinese | English | Chinese | English |
| 调整 | adjust | 表露 | reveal | 创新 | innovate |
| 制止 | restrain | 释放 | release | 完成 | complete |
| 控制 | control | 表达 | express | 浪费 | waste |
| 调控 | regulate | 外显 | exposure | 应酬 | entertain |
| 抑制 | suppress | 展现 | display | 跑步 | run |
| 调节 | regulate | 表现 | show | 检查 | examine |
| 平息 | Calm | 流露 | betray | 覆盖 | cover |
| 克制 | restrain | 显露 | demonstrate | 闪耀 | shine |
| 隐藏 | hide | 外露 | manifest | 后退 | retreat |
| 包容 | tolerate | 展示 | display | 降低 | reduce |
|  |  |  |  | 流浪 | wander |
|  |  |  |  | 复活 | resurrect |
|  |  |  |  | 筹划 | plan |
|  |  |  |  | 锻炼 | exercise |
|  |  |  |  | 取消 | cancel |
|  |  |  |  | 计划 | plan |
|  |  |  |  | 走私 | smuggle |
|  |  |  |  | 挨饿 | starve |
|  |  |  |  | 迟到 | be late |
|  |  |  |  | 考试 | examine |

**2. Pictures used in the facial expression identify task**

The CFAPS identification numbers of the selected pictures were the following for anger: AF1, AF3, AF4, AF5, AF9, AF13, AF14, AF15, AF17, AF18, AF19, AF20, AF26, AF28, AF29, AF30, AF32, AF33, AF34, AF35, AM1, AM4, AM6, AM8, AM9, AM10, AM11, AM13, AM14, AM16, AM17, AM18, AM19, AM20, AM21, AM26, AM27, AM28, AM33, AM37; for fear: FF1, FF2, FF4, FF6, FF7, FF8, FF11, FF13, FF14, FF15, FF17, FF18, FF19, FF20, FF21, FF22, FF23, FF24, FF26, FF27, FM1, FM3, FM4, FM6, FM7, FM9, FM11, FM16, FM21, FM23, FM25, FM26, FM28, FM29, FM30, FM32, FM33, FM34, FM35, FM36.
